# Beyond surgery: bladder preservation and the role of systemic treatment in localised muscle-invasive bladder cancer

**DOI:** 10.1007/s00345-024-04892-8

**Published:** 2024-04-04

**Authors:** Martin Swinton, Aarani Devi, Yee Pei Song, Peter Hoskin, Ananya Choudhury

**Affiliations:** 1 Christie Hospital NHS Foundation Trust, Manchester, UK; 2 University of Manchester, Manchester, UK

**Keywords:** Bladder preservation, Bladder cancer, Immunotherapy, Hypoxia

## Abstract

Over the last two to three decades the non-surgical curative management of bladder cancer has significantly progressed. Increasing evidence supports the use of bladder preservation as an alternative to radical cystectomy (RC) for localised muscle-invasive bladder cancer (MIBC). Radiosensitisation with chemotherapy or hypoxia modification improves the efficacy of radiotherapy. Systemic treatments play an important role in the management of localised MIBC with the benefit of neoadjuvant chemotherapy prior to radical treatment well established. The use of immune checkpoint inhibitors (ICIs) in the radical treatment of bladder cancer, their safe combination with radical radiotherapy regimens and whether the addition of ICIs improve rates of cure are outstanding questions beginning to be answered by ongoing clinical trials. In this narrative review, we discuss the current evidence for bladder preservation and the role of systemic treatments for localised MIBC.

## Introduction

Radical cystectomy (RC) had traditionally been considered the standard curative-intent treatment for muscle-invasive bladder cancer (MIBC) [1]. It is a major surgical procedure that can be associated with significant morbidity [2]. There is increasing evidence that a bladder-sparing treatment approach is equivalent to RC in terms of disease control [3]. Bladder preservation—or trimodality therapy (TMT)—combines maximal transurethral resection of the bladder tumour (TURBT), followed by radiotherapy with concurrent radiosensitisers. Addition of radiosensitisers improved clinical outcomes compared to radiotherapy (RT) alone, leading to its acceptance in clinical guidelines for management of MIBC patients [4].

In this comprehensive review, we discuss the evidence for bladder preservation in localised MIBC and the use of systemic treatments with chemotherapy and immunotherapy in the neoadjuvant, concurrent and adjuvant settings.

## Radiotherapy versus surgery

The traditional view that RC is the sole gold standard for radical treatment of MIBC does not hold up against recent data. Increasing evidence has demonstrated trimodality therapy to be an equivalent, possibly even superior treatment to RC with regards to disease control [3]. Long-term follow-up from trials of radiotherapy combined with a radiosensitiser show overall survival (OS) rates equivalent to RC series of around 50% at 5 years [5].

A randomised control trial (RCT) directly comparing RC to a bladder-preservation protocol was attempted but failed to recruit sufficiently. This was due to a low rate of patients accepting randomisation and a lack of equipoise amongst recruiting clinicians [6, 7].

However, several retrospective analyses of patient cohorts with matched baseline characteristics receiving either RC or TMT have shown equivalent outcomes. A large recently published series of 722 North American patients treated between 2005 and 2017 used 2 independent statistical analyses with propensity score matching to compare outcomes for patients receiving either RC (*n* = 440) or TMT (*n* = 282) [3]. Both statistical methods showed equivalent rates of metastasis-free survival and improved 5-year OS with TMT versus RC. Recruited patients had cT2-T4N0M0 BC and were suitable for either treatment. These results are consistent with previous series showing equivalent outcomes between RC and TMT cohorts when patients’ pre-treatment characteristics are matched [8, 9]. Equivalent outcomes between RC and TMT have also been seen in patients presenting with clinical node-positive MIBC [10].

This evidence implies that both RC and bladder-preservation with trimodality therapy should be presented as options to patients with MIBC to allow them to make an informed choice.

## Radiotherapy technique

Optimisation of radiotherapy comes from both technological advances in radiation delivery and employing knowledge of bladder cancer (BC) biology and radiobiology to guide selection of radiotherapy dose regimen and treatment field.

Advances in the technical delivery of radiotherapy [from a 4-field box technique to 3D conformal radiotherapy (3D-CRT), intensity modulated radiotherapy (IMRT) and volumetric modulated arc therapy (VMAT)] have allowed a greater ability to shape areas receiving high dose radiation; better matching delivered dose to the target tumour and avoiding dose to adjacent organs to reduce radiation toxicity [11]. Image-guided radiotherapy can capture variation in bladder filling and organ motion across fractions allowing smaller radiation volumes without risking tumour being missed.

Recent evidence supports the use of hypofractionation over a conventional regimen to radically treat MIBC [12]. Within the UK, there are two dose regimens commonly used to radically treat localised MIBC [13]; a conventionally fractionated regimen (2 Gy per fraction, 64 Gy in 32# over 6½ weeks) and a hypofractionated regimen (2.75 Gy per fraction, 55 Gy in 20# over 4 weeks). Proponents of conventional fractionation argue the expected high *α*/*β* ratio of BC predicts lower dose per fraction should lead to a better therapeutic ratio between late toxicity rates and tumour control probability. However, the results of meta-analysis of individual patient data from the BC2001 and BCON trials where both dose regimens were permitted, showed superior locoregional control with hypofractionation and comparable toxicity rates [12]. These results suggest that both the *α*/*β* ratio of BC is lower and tumour repopulation more important than had been predicted. Fewer fractions of radiotherapy also translates to greater convenience for patients and lower costs of treatment, all favouring the adoption of a hypofractionated regimen.

Delivering a uniform tumoricidal dose to the whole bladder with a 1.5-cm isotropic expansion to the planned target volume (PTV) to compensate for changes in bladder volume is standard practice in the UK [13] and was used in both BC2001 and BCON trials [14, 15]. The RAIDER trial investigates a modification to this, using a ‘plan of the day’ image-guided radiotherapy strategy. One therapeutic arm reduces dose in the non-tumour bladder (standard dose adaptive tumour focused radiotherapy—SART). A second arm escalates dose to tumour and reduces dose in non-tumour bladder (dose escalated adaptive tumour boost arm—DART). Patients receive either standard fractionation or hypofractionation. Results presented to date have shown DART to meet the primary outcome of a < 20% G3 toxicity. The trial was not powered for comparison of efficacy, but there is a suggestion of a better bladder intact survival with DART [16].

**Fig. 1:**
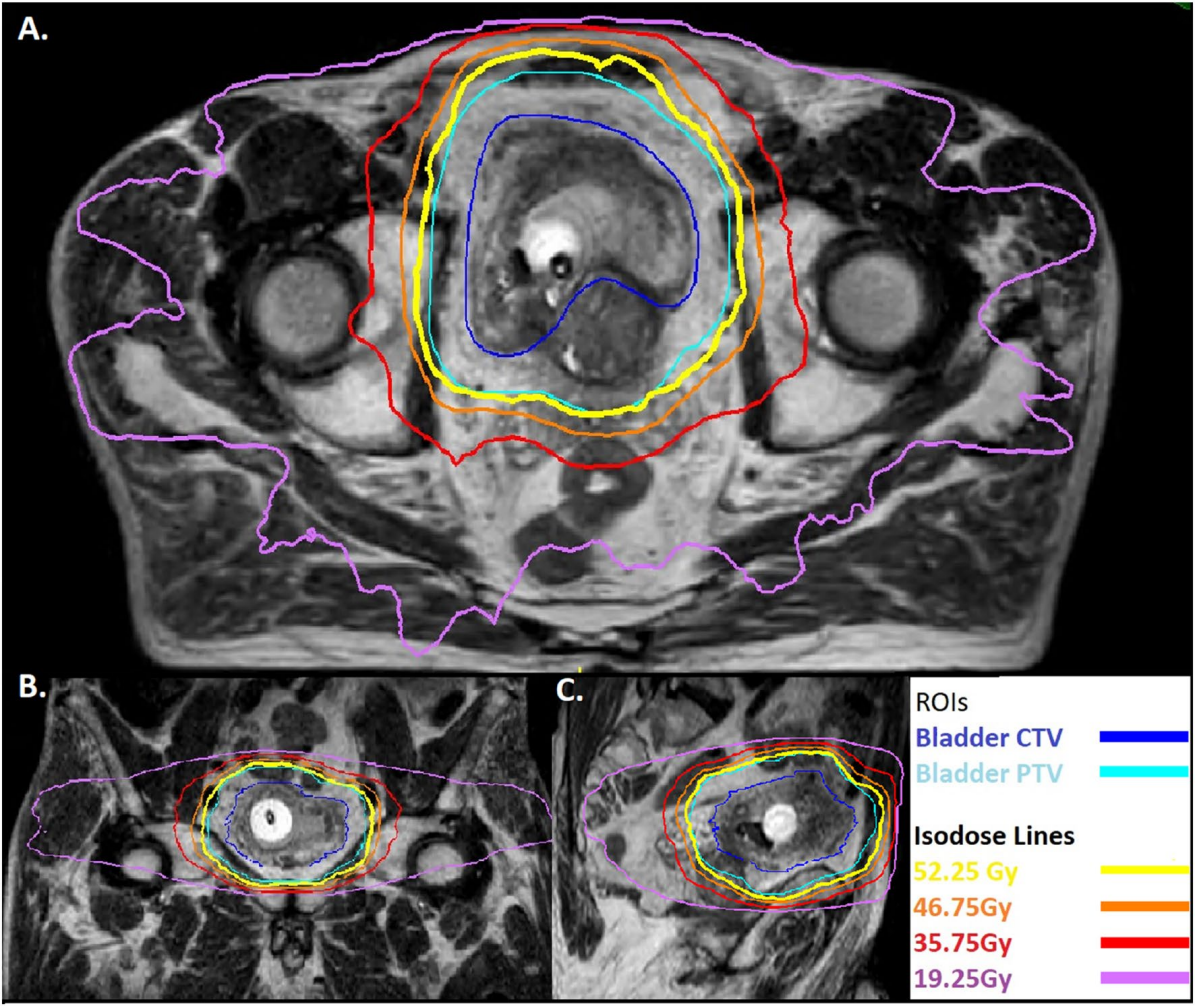
Patient with urothelial cell carcinoma of the bladder and a long-term urinary catheter receiving 55 Gy in 20 fractions of radiotherapy with concurrent BCON (carbogen and nicotinamide). Images acquired on the MR Linac with axial (**A**), coronal (**B**) and sagittal (**C**) views shown. Bladder Clinical Target Volume (CTV, royal blue) is expanded to bladder planning target volume (PTV, light blue). Isodose lines from the radiotherapy plan are shown with good conformality of the 95% isodose line (52.25 Gy, yellow) around bladder PTV

Daily imaging identifies changes in organ size, shape and position between fractions; allowing margins to be reduced without missing the bladder [17]. The MR Linac integrates a magnetic resonance (MR) scanner with a linear accelerator (Linac) that delivers radiotherapy. It allows on treatment imaging with better differentiation of soft tissue structures than a conventional Linac (Fig. 1) potentially allowing further reduction of margins. In addition, newer techniques of radiation delivery such as VMAT allow better ‘conformality’ of the high-dose region around the intended target. Before reducing treatment field though, a potential contribution of ‘incidental dose’ delivered to surrounding lymph nodes on disease control should be considered. Rates of occult lymph node metastases of around 25% have been seen in surgical series in patients staged as N0 on imaging [18], but the rate of pelvic node relapse in BC2001 (in which radiotherapy was delivered to bladder only and pelvic lymph nodes not treated) was only 5.8% (21/360) [14]. An explanation for this disparity could be the incidental delivery of clinically meaningful radiation dose to adjacent lymph nodes with bladder-only techniques [19]. Elective pelvic nodal radiotherapy has failed to demonstrate benefit in BC [20] which again could be due to incidental dose to lymph nodes with bladder-only radiotherapy. Therefore, reduction in expansion margins around the bladder should be performed with caution.

## Radiosensitisation: the role of chemotherapy agents

The addition of concurrent chemotherapy to radiotherapy improves disease outcomes compared to bladder RT alone [14]. By combining chemotherapy agents with radical RT, a synergistic effect is achieved, targeting radioresistant cells and promoting increased cell death. Initially, cisplatin was the primary radiosensitiser used, either alone or in combination with 5-FU or paclitaxel. While this approach reported improved response rates and comparable long-term disease-specific survival to RC, the risk of renal toxicity in MIBC patients with impaired renal function and other comorbidities was a major concern [21].

The BC2001 trial compared radiotherapy with concurrent 5-FU and mitomycin C to radiotherapy alone. Better rates of locoregional control were seen in the chemoradiotherapy group with a 5-year locoregional recurrence-free rates of 63% versus 49% (hazard ratio [HR] 0.61, 95% CI 0.43–0.86) [22] after a median follow-up of 10 years.

Single-agent gemcitabine has shown promising results in several prospective trials. An individual patient meta-analysis assessing concurrent gemcitabine and radiotherapy reported a 93% complete response rate at 12 weeks and a 5-year OS rate of 59%, further highlighting the potential of this approach in MIBC management [23].

In a phase 2 trial which compared patients who received twice daily radiation with concurrent fluorouracil plus cisplatin to once daily radiation with concurrent gemcitabine, primary end point of freedom from distant metastasis at 3 years (DMF3) was comparable between both arms (> 75% in both arms). There were fewer toxicities observed in the gemcitabine arm [24].

While the benefits of adding a concurrent chemotherapy agent to radiotherapy compared to radiotherapy alone have been established [14, 24] there is a lack of trial data or consensus on the optimum chemotherapy agent to choose [25] with variation in practice based on patient factors, chemotherapy agent availability as well as individual clinician preference.

## Radiosensitisation: hypoxia modification

Tumour hypoxia is a poor prognostic factor as it can result in genetic instability, radioresistance and chemoresistance. Hypoxia modification, using concurrent carbogen (2% CO_2_ and 98% O_2_) and nicotinamide with RT is an accepted standard treatment in the UK for localised MIBC [4]. Nicotinamide is a derivative of Vit B3 and has an effect on tumour blood flow and reduces acute hypoxia [26].

In the BCON trial [15] investigated the benefit of concurrent hypoxic modification with radiotherapy. Patients were randomised (1:1) to radiotherapy with or without hypoxia modification with carbogen at 15 L/min for 5 min before and during each fraction and nicotinamide (orally at 40–60 mg/kg, given 1.5–2.0 h before each fraction). Addition of BCON improved local control and demonstrated a 13% absolute OS benefit at 3 years (59% versus 46%, *p* = 0.04) with no reported increase in late toxicity. The presence of tumour necrosis, a high hypoxia gene score and a basal molecular subtype were all associated with a greater benefit from the addition of BCON [5].

The identification of hypoxic tumours holds immense promise in refining therapeutic strategies and increasing survival rates. Histopathological markers like necrosis, and biomarkers like hypoxia-inducible factor 1α (HIF-1α), glucose transporter 1 (GLUT-1), and carbonic anhydrase IX (CAIX) to assess protein expression, microRNAs, and novel mRNA signatures, can accurately pinpoint hypoxic regions within tumours. Integrating these into routine clinical practice can empower clinicians to tailor treatments based on tumour biology.

Future interventional trials should emphasise biomarker stratification to identify patients most likely to benefit from hypoxia-modified RT. This tailored approach can improve treatment responses and redefine standards of care for patients with MIBC.

## Role of neoadjuvant chemotherapy

Neoadjuvant treatment targets micro-metastatic disease potentially improving disease-free survival (DFS) and OS as well as offering the potential for downsizing primary tumour volume, particularly in cases where transurethral resection of bladder tumour (TURBT) is incomplete. The BA06 trial allowed for either surgery or radiotherapy following cisplatin, methotrexate, and vinblastine (CMV) administration, reporting a 16% reduction in the risk of death and a favourable 3-year survival increase from 50 to 56% in favour of neoadjuvant chemotherapy [27]. An interaction analysis did not show any evidence that the benefit from neoadjuvant chemotherapy differed between the type of radical treatment received, suggesting the benefit was present regardless of whether the patient had surgery or radiotherapy. Platinum-based combination chemotherapy before RC or radiotherapy has shown promise in providing absolute OS benefits of 5% and DFS benefits of 9% at 5 years [28]. However evidence is limited about its role before chemoradiotherapy. In BC2001 only 31.3% of patients received neoadjuvant chemotherapy before chemoradiation [22]. There was an indication that DFS was better in patients receiving neoadjuvant chemotherapy independent of the use of concurrent chemotherapy, but the study was not powered to detect a statistically significant difference.

Neoadjuvant chemotherapy is considered standard treatment as a part of bladder preservation in the UK [4]. Chemotherapy regimens in use include gemcitabine and cisplatin (GC) and dose-dense methotrexate, vinblastine, doxorubicin, and cisplatin (dd-MVAC). These were compared within the VESPER trial [29] which enrolled 493 patients with non-metastatic MIBC. Patients were randomly assigned to receive either six cycles of ddMVAC or four cycles of GC either before or after RC. The ddMVAC arm showed improved 3-year progression-free survival (PFS) compared to GC (64% versus 56%, hazard ratio (HR) = 0.77 (95% CI 0.57–1.02), and improved time to progression (TTP; 69% versus 58%, HR = 0.68 (95% CI 0.50–0.93). However, ddMVAC was associated with higher grade ≥ 3 toxicity, including gastrointestinal toxicities and asthenia, leading to only 60 percent of patients completing the planned six cycles of treatment due to toxicity issues.

In a separate randomised phase II SWOG S1314 trial [30], involving 237 patients with MIBC, similar pCR rates were observed for ddMVAC compared with GC (28 versus 30 percent, *p* = 0.75). Preliminary results also indicated similar OS and PFS rates between the two chemotherapy regimens.

Thus ddMVAC has demonstrated activity in the neoadjuvant treatment of MIBC, shortening the time to surgery and making it a reasonable treatment option, especially for young patients with good performance status and no comorbidities. Carboplatin is not considered an optimal therapy compared to cisplatin-based chemotherapy as there are no randomised data supporting its role in the neoadjuvant setting. Patients with kidney function impairment may be offered modified regimens, such as GC plus split-dose.

## Role of adjuvant chemotherapy

The role of adjuvant cisplatin-based chemotherapy following cystectomy remains unclear. The role of adjuvant chemotherapy after combined-modality, bladder-sparing therapy has not been examined in prospective controlled studies. Multiple adjuvant cisplatin-based combinations have been studied in randomised trials but studies have not met accrual goals, limiting OS analysis. The ABC meta-analysis, which included 10 trials, found an OS benefit for cisplatin-based chemotherapy (HR, 0.82; 95% CI 0.70–0.96) with an absolute adjusted improvement in 5-year OS of 9%. However, this meta-analysis included heterogeneous studies with poor accrual across a wide timeframe (1984 to 2014). Due to the lack of evidence for adjuvant chemotherapy, it is not considered to be standard of care [31].

## Immunotherapy with RT

Immune checkpoint inhibitors (ICI)s modulate the immune response to cancer cells by targeting programmed cell death 1 receptor (PD-1), its ligand (PD-L1) or cytotoxic T lymphocyte antigen 4 (CTLA-4). Adding ICIs as a ‘fourth modality’ [32] to trimodality treatment for localised MIBC is currently being investigated in ongoing phase II and phase III clinical trials [1]. This is in part driven by a clinical need for treatment intensification in this setting. Outcomes from bladder radiotherapy with a radiosensitiser are equivalent to RC [3] but around half of treated patients will have died within 5 years often as a result of metastatic disease [5, 22].

There is optimism that ICIs combined with radiotherapy may be effective following their successful use in non-small cell lung cancer (NSCLC), where the addition of adjuvant durvalumab to chemoradiotherapy has shown an OS benefit [33]. ICIs have approval and are in widespread use in the metastatic setting for BC indicating an efficacy of these drugs against urothelial cell carcinoma which may translate into a benefit in the radical setting. Finally, there is pre-clinical evidence that radiotherapy and ICIs might have a synergistic effect with radiotherapy priming an immune response through release of tumour antigens which has been postulated to explain the occasionally observed off-target effects or ‘abscopal’ of radiotherapy [34, 35].

However, there are reasons for caution. In NSCLC, use of ICIs showed very clear survival benefit in the metastatic setting both against chemotherapy and as an addition to chemotherapy, with the PD-L1 biomarker clearly defining a group enriched for responders. In contrast, the evidence for ICIs in metastatic BC is less definitive with two large negative phase 3 trials [36, 37] coming after early FDA approval based on phase 2 results and a biomarker selected group of responders being more difficult to identify in BC. Toxicity is also a concern when adding the treatment concurrently to radiotherapy plus a radiosensitiser.

The first high-quality evidence of whether or not the addition of ICIs to radiotherapy leads to better outcomes should come from two Phase III trials currently recruiting participants [38, 39]. The Keynote-992 trial [38] adds pembrolizumab to radical radiotherapy and the INTACT trial [39] adds atezolizumab. BL-13, an international phase II trial is investigating the addition of adjuvant durvalumab to trimodality treatment [40].

To answer the question of whether combining ICIs with radiotherapy is well tolerated there is data on safety and toxicity from Phase I and II trials and an early analysis from INTACT [1]. The early results paint a mixed picture of the toxicity and tolerability of RT + ICI with marked variation across trials. Two Phase I trials were halted due to dose-limiting toxicity [17], but in other studies toxicities rates have been far lower [39, 41].

Two Phase I trials combining RT and ICIs had high level of toxicity. Marcq et al. [42] recruited 8 patients with MIBC to receive radiotherapy to bladder (50 Gy in 20#) and pelvic nodes (40 Gy in 20#) plus gemcitabine (100 mg/m^2^ weekly for 4 weeks) and atezolizumab from day 1 of radiotherapy. Overall, 4 patients had grade 3 colitis requiring hospital admission leading to the trial to be stopped. The PLUMMB trial also stopped early due to toxicity after combining hypofractionated RT with immunotherapy. In PLUMMB, radiotherapy was given at 36 Gy across 6 weekly fractions with pembrolizumab. In the 5 recruited patients, 2 had dose-limiting toxicity (DLT) Grade 3/4 toxicity [18].

In contrast treatment was better tolerated in other trials. The INTACT trial has published a safety analysis after the first 73 patients. While there were higher rates of grade 3 toxicity in the atezolizumab arm compared to control (62% versus 31%), these were mostly haematological. One patient in the atezolizumab arm had grade 3 radiation cystitis but no patients had grade 3 or worse colitis [39]. The CRIMI trial looked at the combination of radiotherapy to the primary tumour (55 Gy in 20#) and pelvic nodes (40 Gy in 20#) with chemotherapy (MMC and capecitabine) plus ICI [41]. Three different immunotherapy regimens were used with both nivolumab 480 mg alone and nivolumab 3 mg/kg plus ipilimumab 1 mg/kg tolerated well while ipilimumab 3 mg/kg with nivolumab 1 mg/kg was stopped after 3 of 6 patients experienced DLT.

Across trials reporting toxicity, there is considerable heterogeneity in treatment given with differences in the ICI used, fractionation regimen, radiosensitiser used and whether radiation field was bladder alone or included pelvic lymph nodes which makes it challenging to identify what the cause of higher toxicity is. Explanations for higher toxicity in Marcq et al. have included the covering of pelvic lymph nodes; however, this was tolerated well in the CRIMI trial. The use of gemcitabine versus MMC/capecitabine between Marcq et al. and CRIMI is another possible difference in tolerability of the regimen.

A concern in the investigation of immunotherapy is that some trials have neglected the use of treatments with established benefit such as BCON [43]. That we can be adding experimental treatments such as ICI without first ensuring the implementation of those with proven efficacy first highlights a concern that evidence-based practice is only implemented when convenient [44].

## Conclusion

Bladder-sparing treatment of localised MIBC has significantly advanced with increasing precision of radiotherapy delivery, addition of radiosensitising agents, benefits from neoadjuvant chemotherapy with the possibility of further benefits with addition of ICI. Evidence now supports it as a valid alternative treatment option to RC for patients suitable for radical treatment. There is evidence to suggest personalising treatments to specific tumour biology could improve outcome. BCs with necrosis or with high levels of hypoxia derived the most benefit from the addition of BCON to radical radiotherapy for example [5]. Biomarker-driven treatment selection, guiding decisions between surgery or bladder preservation, choice of radiosensitiser, choice of radiotherapy field size and the use of dose escalation could potentially improve outcomes by more effectively tailoring each treatment to groups enriched with responders. Prospective trials of different treatment approaches that incorporate candidate predictive biomarkers are needed so that the progress in BC treatments continues in the years ahead.

## Data Availability

Not applicable.
